# Nipah virus outbreak trends in Bangladesh during the period 2001 to 2024: a brief review

**DOI:** 10.1016/j.soh.2024.100103

**Published:** 2024-12-30

**Authors:** Awnon Bhowmik, Mahmudul Hasan, Md. Mehedi Hasan Redoy, Goutam Saha

**Affiliations:** a Colorado State University, Global Campus, 555 17th St., Ste. 1000, Denver, CO, 80202, United States; b University of Dhaka, Dhaka, 1000, Bangladesh; c Miyan Research Institute, International University of Business Agriculture and Technology, Uttara, Dhaka, 1230, Bangladesh

**Keywords:** Nipah virus, Bangladesh outbreaks, Infection trends, Mortality rates, Climatic factors, Northwestern high-risk regions, Public health interventions

## Abstract

Nipah virus (NiV) is a zoonotic threat that has caused recurrent outbreaks in Bangladesh since 2001, raising significant public health concerns. This study provides a descriptive analysis of NiV outbreaks from 2001 to 2024, examining trends in infection and death rates and their correlation with climatic factors such as temperature, humidity, and rainfall. The findings highlight significant spikes in NiV cases during specific years, with environmental factors, particularly temperature and precipitation, showing solid correlations with outbreak patterns. The study also explores the impact of population dynamics on transmission risks, including urbanization and density. By focusing on these factors, this research supports the development of targeted public health interventions in high-risk areas, particularly in Bangladesh's northwestern and central districts, where recurrent outbreaks have been observed. These insights improve surveillance and preventive strategies for mitigating future NiV outbreaks.

## Graphical abstract

**Figure:**
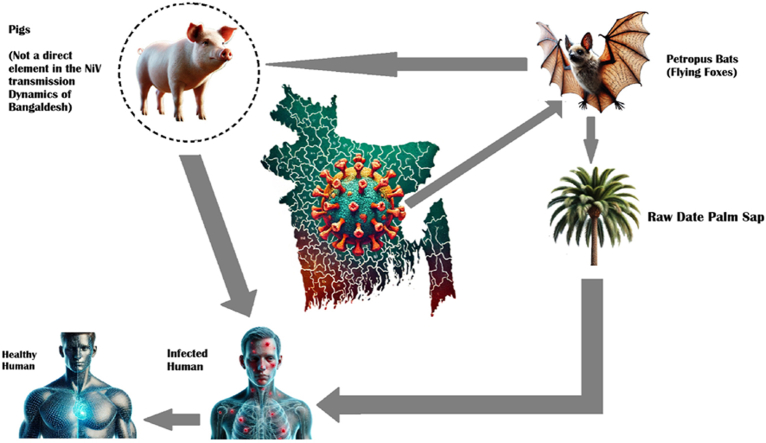

## 1 Introduction

Nipah virus (NiV) is a zoonotic pathogen that has emerged as a significant public health concern in South and Southeast Asia [1,2]. It was first identified during a devastating outbreak among pig farmers in the village of Sungai Nipah, lasting from September 1998 to June 1999 [[3], [4], [5], [6]]. Singapore experienced its effects next in 1999 [7,8]. Since then, NiV has become a recurring issue, particularly in Bangladesh, which has reported almost annual outbreaks since 2001 [9,10]. Initially, its effects were attributed to the Japanese encephalitis virus [11]. However, preliminary studies by the Centers for Disease Control and Prevention (CDC) in the USA revealed that NiV shares ultrastructural, antigenic, serologic, and molecular similarities with Hendra virus (HeV), a virus that emerged in an Australian horse training complex [12].

Epidemiological evidence suggests that the movement of pigs between farms and regions was a key factor in NiV transmission during the initial outbreak [13]. Investigations revealed that the virus had been present in Malaysia since late 1996, but it was not recognized as a new syndrome due to its similarity to several endemic diseases with low infection and death counts [14]. NiV primarily affects endothelial and neuronal tissues, causing serious brain inflammation, which is marked by damage to blood vessels and the central nervous system [[15], [16], [17], [18], [19]]. Usually, the incubation period lasts four to fourteen days [2]. Approximately 20 % of cases during the Malaysia–Singapore pandemic and 70 % of cases in Bangladesh and India exhibited respiratory complications [20,21]. Other symptoms include fever, tiredness, headache, muscle pain, nausea, vomiting, dizziness, and confusion [2,21]. Many patients arrive at the hospital in critical condition, reducing their chances of survival. Early diagnosis could improve outcomes [22].

The main NiV carriers are *Pteropus* bats, sometimes referred to as fruit bats or flying foxes [6,23]. Even though the bats themselves don't exhibit any symptoms, the virus spreads when humans consume raw date palm sap polluted with bat urine or saliva [24,25]. Ecological changes, such as deforestation and habitat fragmentation, are pushing bats closer to human settlements, altering their feeding patterns and increasing the risk of human–bat interactions [[26], [27], [28]]. These changes have led to increased human exposure to contaminated date palm sap, particularly during the winter months when sap harvesting occurs, heightening the risk of transmission [[29], [30], [31], [32]]. Surveys suggest that people who climb trees to harvest sap may be more susceptible to infection [33,34]. Additionally, human-to-human transmission through respiratory droplets and contaminated surfaces has complicated containment efforts [35].

The first documented NiV case in Bangladesh occurred in Meherpur district in 2001, and sporadic cases have since been reported in 32 out of 64 districts. Since 2001, NiV has infected approximately 330 people, claiming 237 lives [36,37]. In Bangladesh, unlike other countries where pigs serve as secondary carriers, fruit bats are the primary carriers, while cows, goats, and pigs have been identified as secondary carriers [38]. The World Health Organization (WHO) has classified NiV as a priority disease due to its high case fatality rate (CFR), potential for human-to-human transmission, and the absence of effective antiviral treatments or vaccines [39,40].

Understanding the drivers of NiV transmission is crucial to preventing future outbreaks [1,13]. Environmental and ecological changes, especially deforestation and human intrusion into wildlife habitats, require attention. Mitigating risk must involve not only medical interventions but also ecological conservation and public health education [2,9]. Collaborative, interdisciplinary approaches will be essential to controlling NiV and reducing its impact on human populations. Ongoing research and global cooperation will play pivotal roles in developing effective strategies for prevention, control, and treatment [13,20].

The aim of this research is to study the outbreak trends of NiV in Bangladesh from 2001 to 2024 by analyzing infection and death data, with the ultimate objective of understanding how the virus's impact on the population has evolved over time. Since NiV was first documented in Bangladesh in 2001, the country has experienced nearly annual outbreaks, with significant fluctuations in infection rates, case fatality ratios, and regional spread. This study seeks to uncover the key factors that have influenced the transmission dynamics and severity of NiV outbreaks, as well as the effectiveness of public health interventions.

## 2 Methods

### 2.1 Data description

The data for this study was gathered from various publicly available sources, including the Institute of Epidemiology, Disease Control, and Research (IEDCR, https://www.iedcr.org/) [41], the WHO (https://www.who.int/), and others. The dataset covers the period from 2001 to 2024 and includes key variables such as the number of infected cases, deaths, and environmental factors like temperature, humidity, and rainfall. These environmental variables were obtained from the Bangladesh Meteorological Department (https://live8.bmd.gov.bd/), which tracks long-term weather patterns across the country. The dataset also includes population dynamics such as urbanization and population density, sourced from the Bangladesh Bureau of Statistics (https://bbs.gov.bd/) and Worldometer, Bangladesh (https://www.worldometers.info/world-population/bangladesh-population/).

### 2.2 Data analysis

Our study's response variables are the discrete count variables of NiV infection and mortality cases. We aim to explore the role of environmental and demographic factors in these responses. To analyze these relationships, we employed statistical methods such as correlation analysis, seasonal decomposition, and explored key metrics like the CFR. This paper primarily focuses on the statistical analysis of the available data, with descriptive statistics used to provide insights into the overall structure of the dataset. However, due to the limited scope of NiV-related data in Bangladesh, we were unable to conduct more complex analyses involving environmental, regional, and weather-related factors. We have used *Python* v3.13 for data preprocessing, *matplotlib* and *seaborn* for plots, *geopandas* for plotting the maps, and *StatsModels* library for performing seasonal decomposition. Our implementations can be reproduced using the code and data made available at our GitHub repository https://github.com/awnonbhowmik/NiV-Data-Analysis.

## 3 Findings and overview

This study delves into the geographical and seasonal patterns of NiV outbreaks in Bangladesh, highlighting the virus's dominance in the northwestern regions and its strong association with specific environmental and ecological factors. By employing statistical decomposition techniques, the analysis reveals key seasonal trends and outbreak fluctuations, offering valuable insights into the virus's behavior over time. The findings underscore the public health challenges posed by NiV, such as its high CFR and sporadic outbreak patterns, while identifying areas for targeted interventions to mitigate future risks.

### 3.1 Geographical and seasonal patterns of NiV infection

**Fig. 1:**
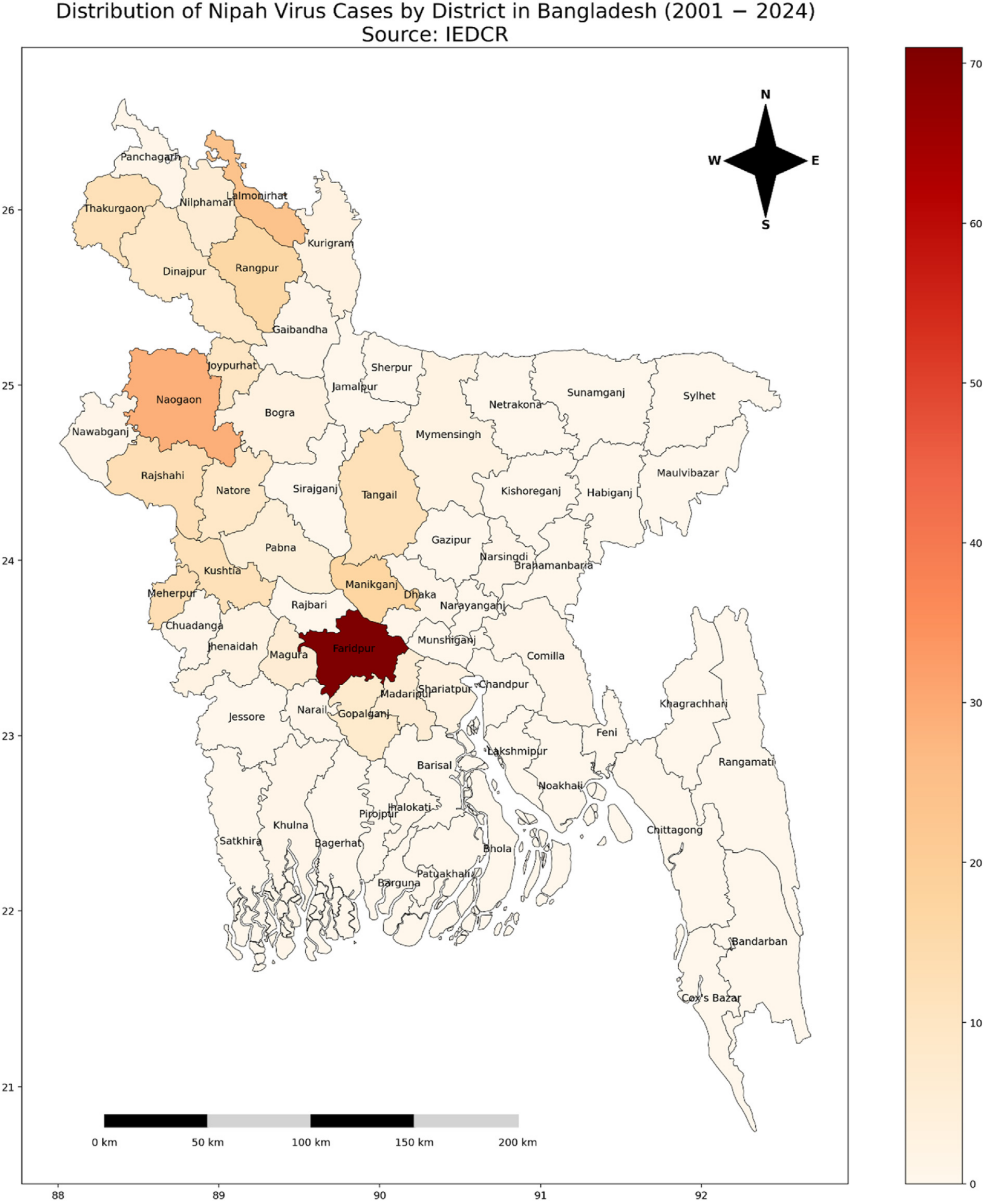
Heatmap of the number of infected cases by NiV in Bangladesh (2001–2024). Data source: https://www.iedcr.org/. Abbreviation: IEDCR, Institute of Epidemiology, Disease Control, and Research.

Fig. 1 shows a heatmap of the number of infected cases by NiV in Bangladesh. It is evident that the disease is dominant in the northwestern region of the country. Despite Rajshahi and Rangpur being a high threat region for the virus, Faridpur district seems to have attained the maximum infected population. This is because date trees are common in the region [42]. Date palm sap is considered a seasonal delicacy, and safety concerns about contamination are overlooked due to its popularity. Date sap is harvested primarily in the winter [43,44], a time when bats are actively feeding on the sap, which increases the chance of contamination during a concentrated time period. This contributes to seasonal spikes in NiV infections. The next regions in line with the most infected population are Naogaon, Lalmonirhat, Manikganj, etc.

### 3.2 Deforestation and its role in NiV surveillance

Deforestation appears to be a significant contributor to the spread of the NiV, as environmental changes, such as loss of forest cover, can disrupt ecosystems and drive wildlife closer to human populations, increasing the risk of zoonotic disease transmission [32,[45], [46], [47], [48]]. However, it is just one of several potential factors that warrant further investigation. According to Global Forest Watch (GFR) (https://www.globalforestwatch.org/dashboards/country/BGD/), Bangladesh lost 246,000 ha of forest cover between 2001 and 2023, representing a 13 % decrease from 2000 levels [49]. The Chittagong region experienced the highest loss, with approximately 231,000 ha of forest cover gone. Other areas with notable deforestation include Sylhet with 8366 ha, Rangpur with 5657 ha, Rajshahi with 323 ha, Khulna with 203 ha, and Barishal with around 99 ha. These regions are consequently designated as active surveillance sites for NiV [50]. Key drivers of deforestation in Bangladesh include overpopulation, illegal logging, and increased reliance on forest and fishing resources for livelihoods. It is worth noting that Rajshahi and Rangpur, two districts mentioned, are located in the northwestern part of Bangladesh, while Sylhet, the second most deforested region, remains under active surveillance despite no reported Nipah cases. Given its high deforestation rates, it's understandable why Sylhet is a focus for monitoring.

### 3.3 Trends and severity of NiV outbreaks

**Fig. 2:**
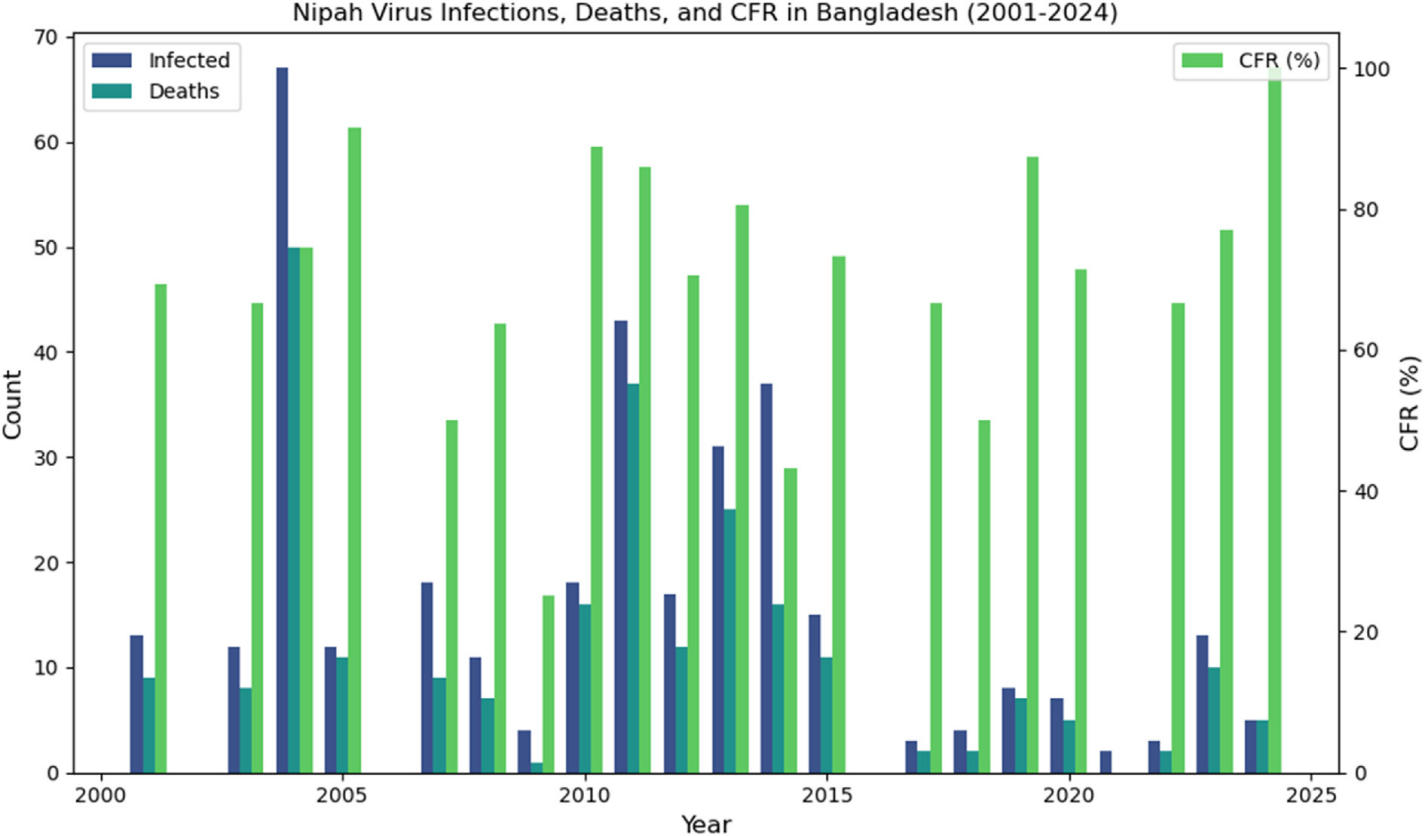
Nipah virus trends in Bangladesh (2001–2024). Abbreviation: CFR, case fatality rate.

The CFR is recorded to be as low as 0 % and as high as 100 % [51]. Fig. 2 shows a trend analysis of the number of infected and deaths caused by the virus between 2001 and 2024. If measured with infected and death counts, major outbreaks occurred in 2004, 2011, 2013, and 2014 with number of infected cases being 65, 43, 30 and 38, respectively. However, when measured with CFR, it is noted to be consistently high, frequently exceeding 50 %, indicating the severity of the virus. The extensive variance in the number of infected and death tolls suggest sporadic outbreak patterns. Despite some years having fewer or no reported cases, the lethal nature of the virus remains significant, underscoring the public health challenges thrown at us.

### 3.4 NiV risk factors and preventive measures

Table 1 summarizes the current state of NiV in Bangladesh, its risk factors and the preventive measures against it. Considering results from survey experiments in 2022, the Nipah season was established as December to the end of April. Communication with the surveillance hospital authority, hospital-level surveillance operations, sample testing, and transportation are all methodically enhanced throughout these months [52]. However, it is important to note that there is no cure or vaccine for this disease. So apparently, discretion is advised for the better part. Preventive measures have been set by the IEDCR [53].

**Table 1:** Current state of Nipah virus (NiV) infection in Bangladesh.

Ref.	Item	Description
	Season	Usually occur between December and April
[54]	Symptoms	Fever, altered mental status, severe weakness, difficulty breathing, vomiting, and cough
[55]	Risk factors	Males are more likely to be infected, and about 40 % of cases are in patients aged 18 or younger
[56]	Treatment	There is no cure or vaccine
[57]	Prevention	Avoid consuming raw date juice or eating fruits that have been partially eaten by bats or birds

### 3.5 Descriptive summary of study data

The dataset used in this study is shown in Table 2. It contains yearly records of NiV infection and death counts. These infection and death counts reported each year are two key variables. The data also contains population dynamics to test whether densely populated areas are susceptible to infection, along with temperature and rainfall statistics. This data is essential for understanding the temporal patterns of NiV outbreaks, analyzing long-term patterns, and assessing the public health impact of the virus in the region. By applying decomposition techniques, we aim to uncover seasonal influences and broader trends that inform outbreak management and mitigation strategies.

**Table 2:** Dataset used for the study.

Year	Pop	*ρ* (population/km^2^)	UPop (population/km^2^)	RPop (population/km^2^)	*T*_max_ (°C)	*T*_min_ (°C)	RF (mm)	Pp (mm)	Hd (%)	*T*_DB_ (°C)	I_NIV_	D_NIV_
2001	136,578,600	1049	32,505,706	104,072,893	33.24	18.28	139.92	2198.86	73.42	25.94	13	9
2002	138,612,896	1065	33,960,159	104,652,737	33.44	17.68	156.25	2127.61	73.17	25.82	0	0
2003	140,647,193	1080	35,443,092	105,204,100	33.52	18.08	141.08	2159.29	73.67	25.75	12	8
2004	142,681,489	1096	36,954,505	105,726,983	33.55	18.02	195.58	2112.04	72.67	25.88	67	50
2005	144,715,786	1112	38,494,399	106,221,386	33.67	18.73	219.75	2092.13	72.83	26.24	12	11
2006	146,213,025	1123	40,033,126	106,179,898	34.62	18.65	159.92	2205.12	71.42	26.47	0	0
2007	147,710,264	1134	41,595,210	106,115,053	33.90	18.29	240.42	2246.41	73.17	25.71	18	9
2008	149,207,503	1146	43,180,651	106,026,851	33.67	18.34	184.75	2384.65	73.42	25.88	11	7
2009	150,704,742	1157	44,789,449	105,915,292	34.66	18.68	160.92	2155.83	70.25	26.52	4	1
2010	152,201,981	1169	46,421,604	105,780,376	34.24	19.12	126.92	2254.19	70.42	26.58	18	16
2011	153,638,220	1180	48,150,218	105,488,002	33.66	18.68	148.00	2384.01	70.92	25.82	43	37
2012	155,074,460	1191	49,902,961	105,171,498	34.12	18.58	110.75	2179.67	70.25	26.09	17	12
2013	156,510,699	1202	51,679,833	104,830,866	34.17	18.40	129.67	2224.81	70.50	26.09	31	25
2014	157,946,939	1213	53,480,833	104,466,105	34.67	18.40	116.58	1971.07	69.83	26.23	37	16
2015	159,383,179	1224	55,305,963	104,077,215	34.36	18.56	180.50	2127.54	70.75	26.16	15	11
2016	160,766,148	1234	57,168,442	103,597,705	34.61	19.52	113.75	2125.41	72.58	26.73	0	0
2017	162,149,117	1245	59,054,708	103,094,408	34.21	19.14	241.00	2384.28	73.42	26.38	3	2
2018	163,532,086	1256	60,964,761	102,567,324	34.32	19.20	144.33	2168.76	71.58	26.08	4	2
2019	164,915,055	1267	62,898,601	102,016,453	34.71	18.46	153.08	2021.15	72.50	26.36	8	7
2020	166,298,024	1278	64,856,229	101,441,794	34.82	19.48	160.00	2186.48	74.83	26.24	7	5
2021	167,841,460	1289	66,800,901	101,040,559	35.40	19.06	156.08	2284.23	71.92	26.78	2	0
2022	169,384,897	1301	68,770,268	100,614,628	33.09	20.20	114.25	2284.23	73.00	27.00	3	2
2023	171,466,990	1317	70,815,866	100,651,123	38.33	21.11	241.57	2284.23	64.00	30.00	13	10
2024	173,562,364	1333	72,896,192	100,666,171	32.78	26.11	350.00	2284.23	75.00	29.44	5	5

Abbreviations: Pop, population; *ρ*, density; UPop, urban population; RPop, rural population; *T*_max_, maximum temperature; *T*_min_, minimum temperature; RF, rainfall; Pp, precipitation; Hd, humidity; *T*_DB_, dry-bulb temperature; I_NIV_, infected; D_NIV_, death.

Table 3 shows the descriptive summary of the dataset used in the study. The minimum and maximum temperatures in the study region are 19.12 °C and 34.24 °C respectively, with an average temperature of 26.51 °C. In addition to serving as an indicator of the level of crowding in the research area, the population density of 377.90 persons per square kilometer can be used to pinpoint regions that are particularly vulnerable to the development of infectious diseases like the NiV.

**Table 3:** Summary of the statistics for different parameters in Bangladesh.

Variable	Mean	Standard deviation	Min	Max
Population	197,728,357.21	11,114,267.97	171,200,000	224,422,497
Density (population/km^2^)	377.90	304.55	231.31	1176.75
Urban population	78,419,350.12	11,114,267.97	61,804,802	98,457,030
Rural population	119,309,007.08	7,101,417.08	103,200,000	125,965,467
Maximum temperature (°C)	34.24	1.07	32.78	38.33
Minimum temperature (°C)	19.12	1.66	17.68	26.11
Rainfall (mm)	170.21	55.53	110.75	350.00
Precipitation (mm)	2201.93	106.51	1971.07	2384.65
Humidity (%)	71.90	2.23	64.00	75.00
Dry-bulb temperature (°C)	26.51	1.05	25.71	30.00
Infected	14.83	15.67	0	65
Death	10.46	11.91	0	48

### 3.6 Analysis of seasonality in NiV outbreaks

**Fig. 3:**
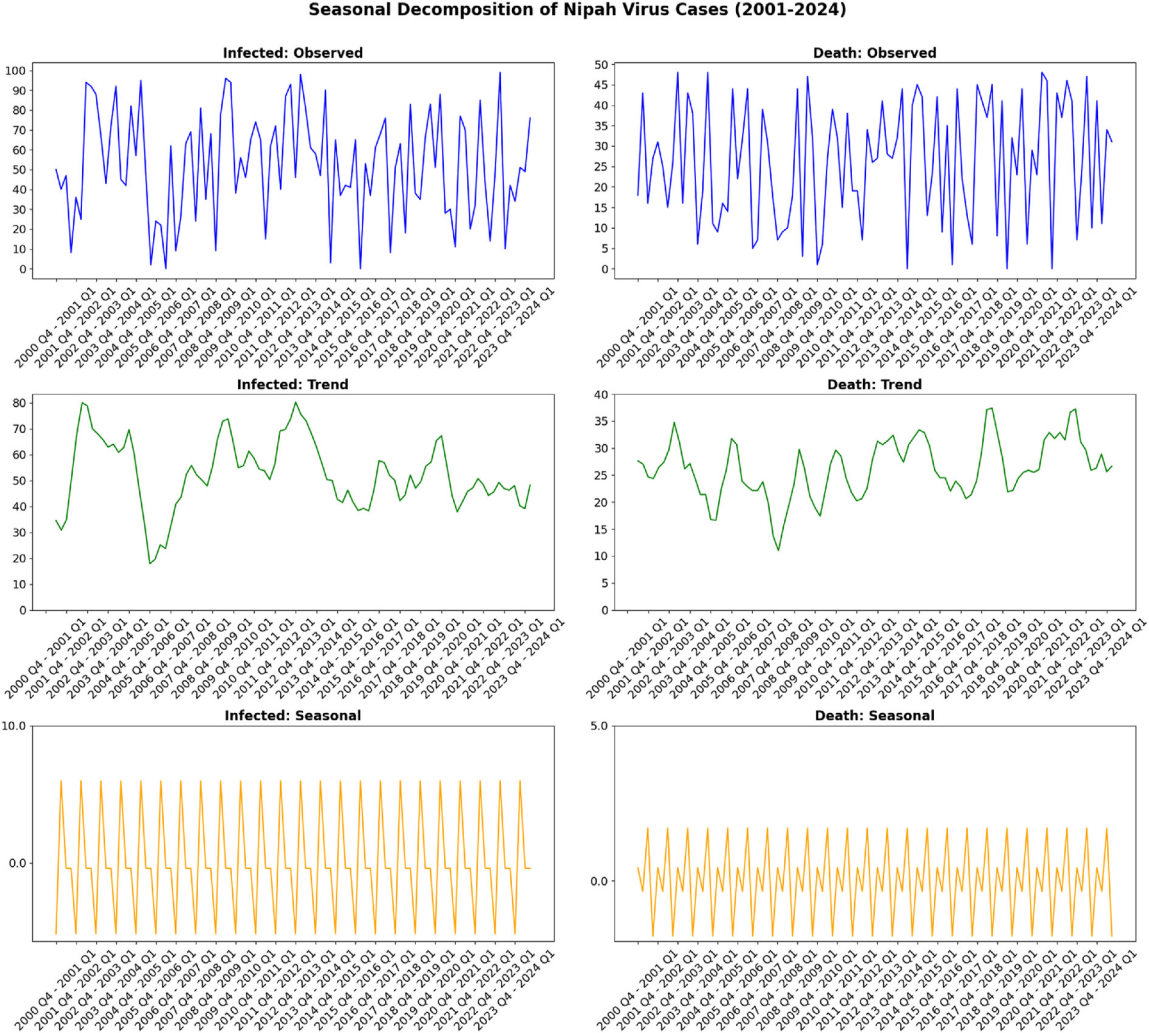
Seasonal decomposition of Nipah virus cases in Bangladesh (2001–2024). Q1: January, February, March; Q2: April, May, June; Q3: July, August, September; Q4: October, November, December.

A time series decomposition was carried out to investigate the presence of seasonality in the working dataset. Fig. 3 shows seasonal decomposition of the infected and death cases.

The decomposition splits the data into three components: observed, infected and death counts. For infected cases, the observed plot shows significant fluctuations over the years, with peaks and troughs reflecting periods of higher and lower infection rates. Trend plot highlights a long-term increase in infections, with a notable upward trajectory from 2001 to 2007, a dip in the mid-2010s followed by another rise towards 2024. The seasonal decomposition reveals a consistent, predictable pattern, indicating periodic increases and decreases within specific quarters, which suggests a seasonal recurrence of outbreaks.

For deaths, the observed plot similarly shows fluctuations but with lower magnitude compared to infections. The trend component indicates a gradual rise in deaths from 2001 to around 2010, followed by a steady decline through the 2020s, possibly reflecting improved healthcare management or reduced severity of outbreaks. The seasonal pattern for deaths aligns with the influence on the virus's spread and impact. The x-axis labels highlight the period of December to March which is a part of Q4 to a part of Q1. This has been established as the Nipah season due to the numerous surveillances.

The significant outbreaks observed in 2004 and 2010, followed by a decline in severity and frequency, indicate that the virus has experienced periods of intense activity followed by stabilization. This pattern reflects the complex interplay of ecological and human factors in driving outbreaks, as highlighted in previous studies [27,29,58]. The lack of seasonal variation underscores the importance of non-seasonal factors in triggering outbreaks, such as changes in human behavior, ecological shifts, or specific environmental events [4,29,[45], [46], [47], [48]].

### 3.7 Environmental trends and NiV transmission

**Fig. 4:**
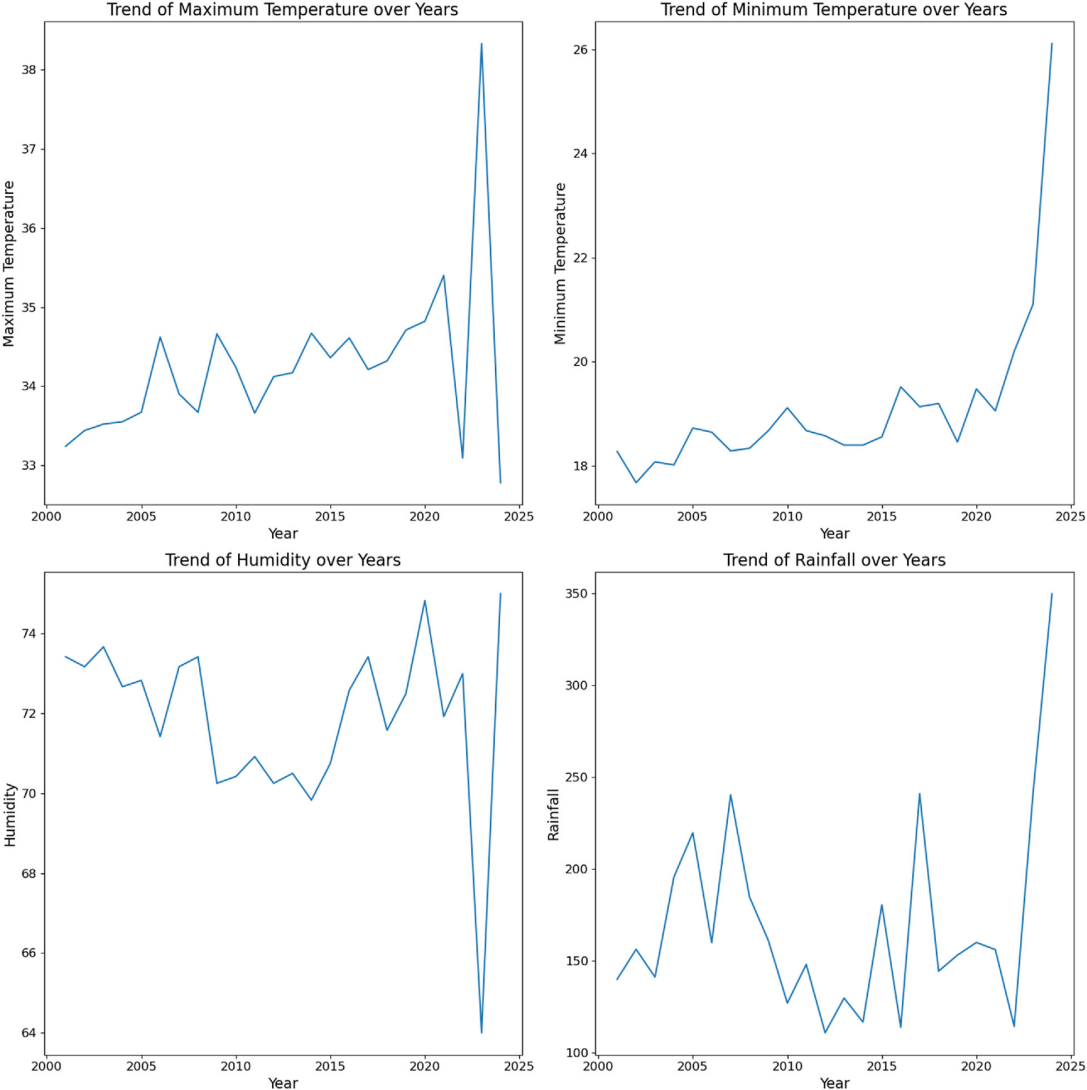
Climatic factor (temperature [°C], humidity [%], and rainfall [mm]) trends over 2001–2024 in Bangladesh.

These four line graphs in Fig. 4 display environmental trends from 2000 to 2024. Both maximum and minimum temperatures show a gradual rise, with a sharp spike in 2024, indicating a warming trend. Humidity exhibits fluctuations with a slight overall decline, followed by a sudden drop in 2024. Rainfall shows variability but experiences a significant increase around 2024. These changes suggest potential climatic shifts that could affect ecosystems, including factors related to NiV transmission, such as bat behavior and population movements.

### 3.8 Pairwise relationships between environmental variables

**Fig. 5:**
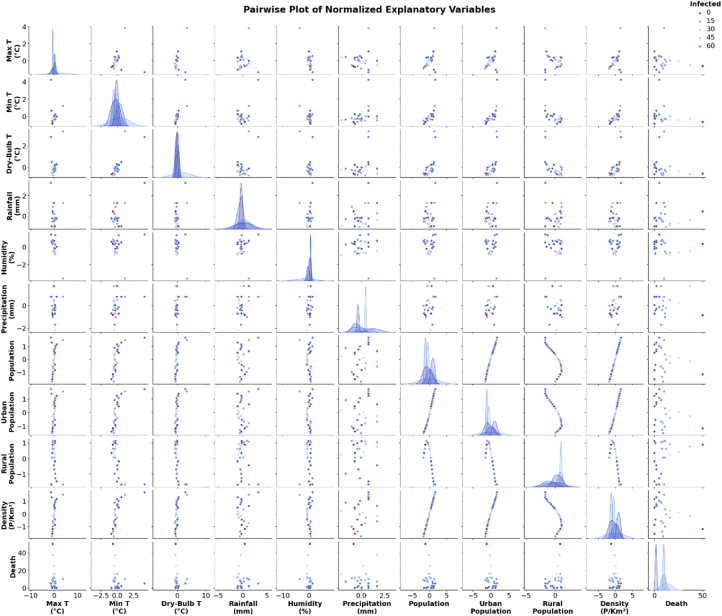
Pairwise scatter plot matrix showing the relationships between climatic and environmental variables, including temperature, humidity, rainfall, and population data. Abbreviations: Dry-Bulb T: dry-bulb temperature; Min T: minimum temperature; Max T: maximum temperature.

This scatter plot matrix in Fig. 5 provides pairwise comparisons of various environmental variables and their relationships to each other. The diagonal contains histograms showing the distribution of individual variables, while the scatter plots reveal any linear or non-linear relationships between pairs of variables. For example, certain variables, like maximum temperature and minimum temperature, show a linear relationship, while others, like precipitation, display more scattered patterns. The curved pattern seen in some variables might suggest non-linear relationships, highlighting potential complexities between factors that could influence NiV transmission or other outcomes.

### 3.9 Correlation analysis of environmental, population, and infection variables

**Fig. 6:**
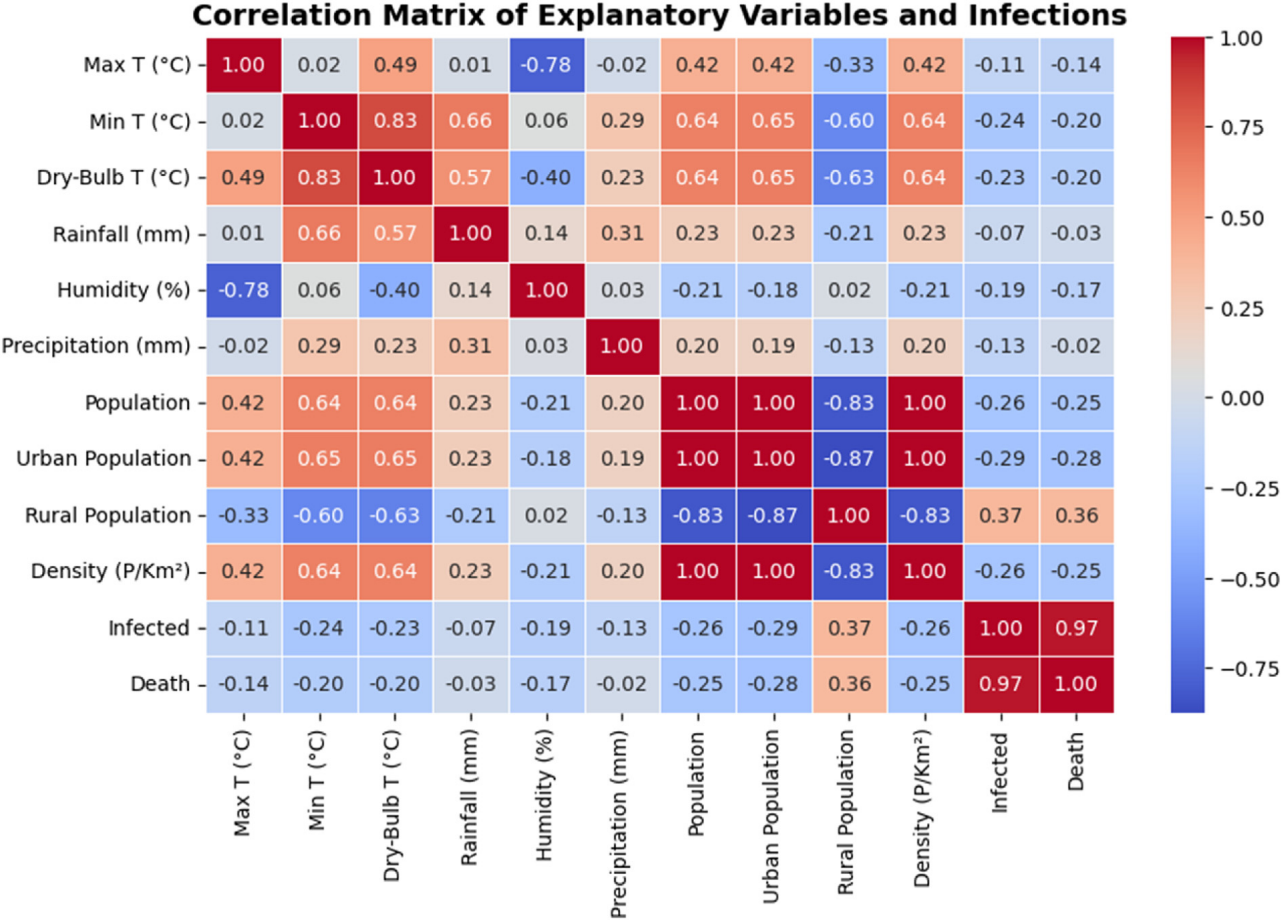
The Pearson Correlation heatmap for influencing variables. Abbreviations: Max T: maximum temperature; Min T: minimum temperature; Dry-Bulb T: dry-bulb temperature.

Fig. 6 presents a correlation matrix of environmental, population, and infection-related variables to understand their relationships with NiV infections and deaths. A strong positive correlation (0.97) between infections and deaths indicates that higher infection rates closely align with mortality. Climate factors like humidity and maximum temperature show a negative correlation (−0.78), while dry-bulb temperature and maximum temperature are positively related (0.49). Additionally, population density (0.64) correlates with minimum temperature, suggesting a potential impact of urbanization on transmission dynamics. Further analysis highlights the moderate correlation between rainfall and minimum temperature (0.66), indicating that areas with higher temperatures experience more rainfall, which could influence bat populations and the likelihood of human exposure to NiV. Similarly, a notable correlation exists between population density and temperature, suggesting urbanization may affect bat habitats and virus transmission. Although correlation does not imply causation, these findings provide valuable insights into the role of environmental and demographic factors in NiV outbreaks.

Population metrics show that the urban population is positively correlated with the total population (0.87) but negatively correlated with both infections and deaths (−0.34), suggesting that the steady increase in urban population may be related to fewer infections. This connection also coincides with the moderate correlation (0.42) between the rural population and total yearly infections. The total rural population has been decreasing annually from 2001 to 2024. A particular case of interest is the year 2023, which has the highest number of infections (13) and deaths (8) since the year 2016. It is also the only year where the total rural population has increased since the year 2005, a change of 0.036 % from 2022 to 2023. The connection between the increase in rural population and the annual NiV infection rate may be explained by the cultural tendency of rural Bangladeshi people to consume raw date palm sap, whereas in urban areas the date palm sap is usually consumed in more refined forms.

### 3.10 Necessary steps towards public health to mitigate future outbreak

NiV outbreaks occur regularly in Bangladesh and Southeast Asia, making early detection and surveillance essential for preventing future outbreaks. Strengthening disease surveillance systems, particularly in rural areas, is key to identifying outbreaks early. Enhancing diagnostic capacity and ensuring real-time data sharing between public health agencies will improve early response. Additionally, continuous surveillance of NiV in endemic countries is crucial for pandemic preparedness [59,60], but it must incorporate adapted diagnostic tests and be aligned with the One Health approach to address the complex interconnections between humans, animals, and the environment [61].

Community education is another vital step. General public must be informed about zoonotic disease risks and promoting safe practices, like avoiding raw date palm sap consumption, can reduce transmission. Engagement programs to raise awareness and encourage early reporting of symptoms will empower communities to participate in prevention.

Investing in vaccine research and development is vital for long-term mitigation of outbreaks like NiV. Governments and global health organizations must collaborate to accelerate vaccine development, as there is currently no cure or approved vaccine for this disease [22,62]. Establishing partnerships for rapid vaccine deployment is also crucial to controlling future outbreaks. Ensuring vaccines are available quickly will significantly enhance global preparedness and response efforts, safeguarding populations from future viral threats.

Strengthening healthcare infrastructure is essential for an effective outbreak response. This involves expanding access to healthcare in high-risk and vulnerable areas, training healthcare workers specifically for epidemic preparedness, and ensuring medical facilities are equipped with isolation units, adequate supplies, and medicine [63,64]. Building robust healthcare systems in these areas will help manage outbreaks efficiently and reduce the risk of further spread by enabling faster responses and better patient care during crises [65].

Environmental and wildlife monitoring is critical in minimizing zoonotic spillovers and preventing outbreaks like NiV. Reducing deforestation and preserving wildlife habitats helps limit human–animal interactions, reducing spillover risks [66]. Monitoring wildlife for early signs of disease and creating guidelines for human-wildlife interactions are essential for reducing transmission [67].

International cooperation is vital for sharing data and coordinating cross-border responses [62]. Strong policies regulating wildlife markets and food safety are necessary to support rapid outbreak responses. Lastly, public health campaigns promoting vaccinations, hygiene, and symptom recognition, using media and technology, are crucial for wider population engagement through technology and media [22].

## 4 Global perspective

The global distribution and impact of NiV reflect its high mortality rates and limited, yet significant, regional outbreaks primarily in South and Southeast Asia. As observed in Table 4, Malaysia's initial outbreak in 1998–1999 resulted in 283 cases with 109 deaths, while Bangladesh has faced almost yearly outbreaks since 2001, totaling 352 cases with a CFR of approximately 74 %. India has also seen irregular outbreaks, such as those in Kerala and West Bengal, with 91 infected and 63 deaths to date. This geographic clustering of outbreaks underscores NiV's persistent threat, particularly in regions with close human-wildlife interfaces. Researchers from the University of Toronto and Vanderbilt University have discussed the likelihood of a NiV outbreak in North America, though currently assessed as low, given the virus's dependency on specific ecological conditions and transmission dynamics [68]. Considering NiV's pandemic potential, global health organizations, including WHO and the Coalition for Epidemic Preparedness Innovations (CEPI), have prioritized it for vaccine research, surveillance enhancement, and preventive strategies. However, ecological drivers like deforestation, which increase zoonotic spillover risks, call for proactive international collaboration in monitoring and preparedness to prevent NiV's spread beyond its present range.

**Table 4:** Nipah virus outbreaks in Southeast Asia (1998–2024) [54,69,70].

Country	Infected cases	Death
Malaysia	283	109
Singapore	11	1
Bangladesh	343	245
India	100	72
The Philippines	17	14

## 5 Limitations and future research

Although this study provides valuable insights on NiV outbreaks in Bangladesh from 2001 to 2024, there are some limitations. With only 24 years of data, predicting long-term trends or building reliable forecasting models is difficult. The random nature of outbreaks and varying infection rates make it challenging to find consistent patterns. Additionally, the analysis focuses on describing trends without using advanced predictive techniques. Future research should expand the dataset and explore more factors to improve the accuracy of predictions and understanding of transmission dynamics.

Due to the limited dataset available for this study, we were unable to conduct a formal correlation analysis regarding deforestation and its potential impact on NiV outbreaks. While deforestation is indeed an important factor that can influence bat migration and human–bat interactions, the data required to perform a robust statistical analysis on this relationship was either incomplete or insufficient in terms of spatial and temporal resolution. We plan to address this limitation in future research, ideally with more detailed data on deforestation patterns and corresponding outbreak occurrences, which would allow for more rigorous statistical analyses.

## 6 Conclusion

In this research, we discussed about NiV and its outbreak in Bangladesh. We talked about deforestation becoming a major reason for bat migration and the subsequent increase in human–bat interaction, thereby affecting the number of people infected by the virus. We discussed how the transmission dynamic of this virus is different than other Southeastern countries, talked about secondary carriers and performed detailed analysis of infection and death trends for the past 24 years. We performed a correlation analysis to gain insights into how climatic factors affect NiV transmission. The sporadic nature of the virus is eminent when seen from a CFR perspective. Moreover, failure of early diagnosis brings infected people to the hospital at critical conditions, reducing their chances of survival. However, we also mentioned about its symptoms being like usual endemics, making it hard for diagnosis. While the virus is seen to be occurring at a specific period in a year (December–April) known as Nipah season, the seasonal decomposition performed didn't show any apparent seasonal trends, due to the data being collected yearly.

## CRediT authorship contribution statement

**Awnon Bhowmik:** Writing – review & editing, Writing – original draft, Visualization, Validation, Software, Resources, Methodology, Investigation, Formal analysis, Data curation. **Mahmudul Hasan:** Writing – original draft, Visualization, Software, Resources, Investigation, Formal analysis. **Md. Mehedi Hasan Redoy:** Writing – original draft, Visualization, Resources, Investigation, Formal analysis. **Goutam Saha:** Writing – review & editing, Writing – original draft, Supervision, Project administration, Conceptualization.

## Ethical approval and consent to participate

Not applicable.

## Availability of data and materials

Not applicable.

## Consent for publication

Not applicable.

## Declaration of Generative AI and AI-assisted technologies in the writing process

The authors used AI-assisted technology (ChatGPT-3.5) for language editing and grammar checking.

## Financial support and sponsorship

None.

## Declaration of competing interest

The authors declare that there are no conflicts of interest.

## References

[1] Hegde S.T., Sazzad H.M.S., Hossain M.J., Alam M.-U., Kenah E., Daszak P. (2016). Investigating rare risk factors for Nipah virus in Bangladesh: 2001–2012. EcoHealth.

[2] Sazzad H.M.S., Hossain M.J., Gurley E.S., Ameen K.M.H., Parveen S., Islam M.S. (2013). Nipah virus infection outbreak with nosocomial and corpse-to-human transmission, Bangladesh. Emerg. Infect. Dis..

[3] Chua K.B., Goh K.J., Wong K.T., Kamarulzaman A., Tan P.S.K., Ksiazek T.G. (1999). Fatal encephalitis due to Nipah virus among pig-farmers in Malaysia. Lancet.

[4] Chua K.B. (2003). Nipah virus outbreak in Malaysia. J. Clin. Virol..

[5] Tan K.S., Tan C.T., Goh K.J. (1999). Epidemiological aspects of Nipah virus infection. Neurol. J. Southeast Asia.

[6] Luby S.P., Rahman M., Hossain M.J., Blum L.S., Husain M.M., Gurley E. (2006). Foodborne transmission of Nipah virus, Bangladesh. Emerg. Infect. Dis..

[7] Paton N.I., Leo Y.S., Zaki S.R., Auchus A.P., Lee K.E., Ling A.E. (1999). Outbreak of Nipah-virus infection among abattoir workers in Singapore. Lancet.

[8] Clayton B.A., Middleton D., Bergfeld J., Haining J., Arkinstall R., Wang L. (2012). Transmission routes for Nipah virus from Malaysia and Bangladesh. Emerg. Infect. Dis..

[9] Chakraborty A., Sazzad H.M.S., Hossain M.J., Islam M.S., Parveen S., Husain M. (2016). Evolving epidemiology of Nipah virus infection in Bangladesh: evidence from outbreaks during 2010–2011. Epidemiol. Infect..

[10] Nikolay B., Salje H., Hossain M.J., Khan A.K.M.D., Sazzad H.M.S., Rahman M. (2019). Transmission of Nipah virus — 14 Years of investigations in Bangladesh. N. Engl. J. Med..

[11] Field H., Young P., Yob J.M., Mills J., Hall L., Mackenzie J. (2001). The natural history of Hendra and Nipah viruses. Microb. Infect..

[12] Murray K., Csiro A.A.H.L., Selleck P., Hooper P., Hyatt A., Gould A. (1995). A Morbillivirus that caused fatal disease in horses and humans. Science (Am. Ass. Adv. Sci.).

[13] Cortes M.C., Cauchemez S., Lefrancq N., Luby S.P., Jahangir Hossain M., Sazzad H.M.S. (2018). Characterization of the spatial and temporal distribution of Nipah virus spillover events in Bangladesh, 2007–2013. J. Infect. Dis..

[14] Aziz J., Olson J., Lee O.B., Daniels P., Adzhar A.B., Bunning M. (1999).

[15] Chadha M.S., Comer J.A., Lowe L., Rota P.A., Rollin P.E., Bellini W.J. (2006). Nipah virus-associated encephalitis outbreak, Siliguri, India. Emerg. Infect. Dis..

[16] Montgomery J.M., Hossain M.J., Gurley E., Carroll D.S., Croisier A., Bertherat E. (2008). Risk factors for Nipah virus encephalitis in Bangladesh. Emerg. Infect. Dis..

[17] Wong K.T., Shieh W.-J., Kumar S., Norain K., Abdullah W., Guarner J. (2002). Nipah virus infection. Am. J. Pathol..

[18] Hsu V.P., Hossain M.J., Parashar U.D., Ali M.M., Ksiazek T.G., Kuzmin I. (2004). Nipah virus encephalitis reemergence, Bangladesh. Emerg. Infect. Dis..

[19] Hassan M.Z., Sazzad H.M.S., Luby S.P., Sturm-Ramirez K., Bhuiyan M.U., Rahman M.Z. (2018). Nipah virus contamination of hospital surfaces during outbreaks, Bangladesh, 2013-2014. Emerg. Infect. Dis..

[20] Gurley E.S., Hegde S.T., Hossain K., Sazzad H.M.S., Hossain M.J., Rahman M. (2017). Convergence of humans, bats, trees, and culture in Nipah virus transmission, Bangladesh. Emerg. Infect. Dis..

[21] Homaira N., Rahman M., Hossain M.J., Epstein J.H., Sultana R., Khan M.S.U. (2010). Nipah virus outbreak with person-to-person transmission in a district of Bangladesh, 2007. Epidemiol. Infect..

[22] Mazzola L.T., Kelly-Cirino C. (2019). Diagnostics for Nipah virus: a zoonotic pathogen endemic to Southeast Asia. BMJ Glob. Health.

[23] Islam M.S., Sazzad H.M.S., Satter S.M., Sultana S., Hossain M.J., Hasan M. (2016). Nipah virus transmission from bats to humans associated with drinking traditional liquor made from date palm sap, Bangladesh, 2011-2014. Emerg. Infect. Dis..

[24] Gurley E.S., Montgomery J.M., Hossain M.J., Islam M.R., Molla M.A.R., Shamsuzzaman S.M. (2007). Risk of nosocomial transmission of Nipah virus in a Bangladesh hospital. Infect. Control Hosp. Epidemiol..

[25] Hossain M.J., Gurley E.S., Montgomery J.M., Bell M., Carroll D.S., Hsu V.P. (2008). Clinical presentation of Nipah virus infection in Bangladesh. Clin. Infect. Dis..

[26] Epstein J.H., Field H.E., Luby S., Pulliam J.R.C., Daszak P. (2006). Nipah virus: impact, origins, and causes of emergence. Curr. Infect. Dis. Rep..

[27] Epstein J.H., Anthony S.J., Islam A., Kilpatrick A.M., Khan S.A., Balkey M.D. (2020). Nipah virus dynamics in bats and implications for spillover to humans. Proceed. Nat. Acad. Sci.- PNAS.

[28] McKee C.D., Islam A., Luby S.P., Salje H., Hudson P.J., Plowright R.K. (2021). The ecology of Nipah virus in Bangladesh: a nexus of land-use change and opportunistic feeding behavior in bats. Viruses.

[29] Plowright R.K., Foley P., Field H.E., Dobson A.P., Foley J.E., Eby P. (2011). Pathways to zoonotic spillover. Nat. Rev. Microbiol..

[30] Khan M.S.U., Hossain J., Gurley E.S., Nahar N., Sultana R., Luby S.P. (2010). Use of infrared camera surveillance to understand bats' access to date palm sap: implications for preventing Nipah virus transmission. EcoHealth.

[31] Jones B.A., Grace D., Kock R., Alonso S., Rushton J., Said M.Y. (2013). Zoonosis emergence linked to agricultural intensification and environmental change. Proceed. Nat. Acad. Sci.- PNAS.

[32] Gavi (2023). https://www.gavi.org/vaccineswork/bangladesh-nipah-virus-keeps-health-system-its-toes.

[33] Gurley E.S., Montgomery J.M., Hossain M.J., Bell M., Azad A.K., Islam M.R. (2007). Person-to-person transmission of Nipah virus in a Bangladeshi community. Emerg. Infect. Dis..

[34] Nazmunnahar, Ahmed I., Roknuzzaman A.S.M., Islam M.R. (2023). The recent Nipah virus outbreak in Bangladesh could be a threat for global public health: a brief report. Health Sci. Rep..

[35] Yadav P.D., Raut C.G., Shete A.M., Mishra A.C., Towner J.S., Nichol S.T. (2012). Detection of Nipah virus RNA in fruit bat (Pteropus giganteus) from India. Am. J. Trop. Med. Hyg..

[36] World Health Organization (WHO) (2024, February 27). https://www.who.int/emergencies/emergency-events/item/2024-e000036.

[37] Urmi T.J., Dewan S.M.R., Rahman J.M., Sharmin S.N., Hassan M.M. (2023). Development of preventive measures and treatment strategies against Nipah virus is a timely need: Bangladeshi perspective. Clin. Pathol..

[38] Chowdhury S., Khan S.U., Crameri G., Epstein J.H., Broder C.C., Islam A. (2014). Serological evidence of Henipavirus exposure in cattle, goats, and pigs in Bangladesh. PLoS Neglected Trop. Dis..

[39] World Health Organization (WHO) (2020). https://www.who.int/news-room/fact-sheets/detail/nipah-virus.

[40] Rahman M., Chakraborty A. (2012). Nipah virus outbreaks in Bangladesh: a deadly infectious disease. WHO South-East Asia J. Publ. Heal..

[41] Institute of Epidemiology, Disease Control and Research (IEDCR) Nipah situation dashboard. https://www.iedcr.gov.bd/site/page/d5c87d45-b8cf-4a96-9f94-7170e017c9ce.

[42] Nahar N., Mondal U.K., Sultana R., Hossain M.J., Khan M.S.U., Gurley E.S. (2013). Piloting the use of indigenous methods to prevent Nipah virus infection by interrupting bats' access to date palm sap in Bangladesh. Health Promot. Int..

[43] Nahar N., Sultana R., Gurley E.S., Hossain M.J., Luby S.P. (2010). Date palm sap collection: exploring opportunities to prevent Nipah transmission. EcoHealth.

[44] Bruno L., Nappo M.A., Ferrari L., Di Lecce R., Guarnieri C., Cantoni A.M. (2022). Nipah virus disease: epidemiological, clinical, diagnostic and legislative aspects of this unpredictable emerging zoonosis. Animals.

[45] Wolfe N.D., Daszak P., Kilpatrick A.M., Burke D.S. (2005). Bushmeat hunting, deforestation, and prediction of zoonoses emergence. Emerg. Infect. Dis..

[46] Daszak P., Cunningham A.A., Hyatt A.D. (2000). Emerging infectious diseases of wildlife-- threats to biodiversity and human health. Science.

[47] Field H.E. (2009). Bats and emerging zoonoses: Henipaviruses and SARS. Zoonoses Publ. Heal..

[48] Pulliam J.R.C., Epstein J.H., Dushoff J., Rahman S.A., Bunning M., Jamaluddin A.A. (2012). Agricultural intensification, priming for persistence and the emergence of Nipah virus: a lethal bat-borne zoonosis. J. R. Soc. Interface.

[49] Ahmad R. (2024). Bangladesh losing forest resources faster than ever. Dhaka Tribune.

[50] Precision Vaccinations (2023, February 20). https://www.precisionvaccinations.com/2023/02/20/nipah-virus-outbreak-73-fatal.

[51] Khan S., Akbar S.M.F., Mahtab M.A., Uddin Md.N., Rashid Md.M., Yahiro T. (2024). Twenty-five years of Nipah outbreaks in Southeast Asia: a persistent threat to global health. IJID Reg..

[52] Satter S.M., Aquib W.R., Sultana S., Sharif A.R., Nazneen A., Alam M.R. (2023). Tackling a global epidemic threat: Nipah surveillance in Bangladesh, 2006–2021. PLoS Neglected Trop. Dis..

[53] Rahman M., Husain M.M. (2011). https://www.iedcr.org/pdf/files/nipah/National_Nipah.pdf.

[54] Aditi, Shariff M. (2019). Nipah virus infection: a review. Epidemiol. Infect..

[55] Tan F.H., Sukri A., Idris N., Ong K.C., Schee J.P., Tan C.T. (2024). A systematic review on Nipah virus: global molecular epidemiology and medical countermeasures development. Virus Evol..

[56] University of Oxford (2024, January 11). https://www.ox.ac.uk/news/2024-01-11-first-human-vaccine-trial-deadly-nipah-virus-launched.

[57] International Society for Infectious Diseases (2019, February). https://isid.org/guide/pathogens/nipah-virus/.

[58] Rahman M.A., Hossain M.J., Sultana S., Homaira N., Khan S.U., Rahman M. (2012). Institute of Medicine (US), *Improving Food Safety through a One Health Approach: Workshop Summary* (A12).

[59] Garbuglia A.R., Lapa D., Pauciullo S., Raoul H., Pannetier D. (2023). Nipah virus: an overview of the current status of diagnostics and their role in preparedness in endemic countries. Viruses.

[60] Sharif N., Sharif N., Khan A., Dey S.K. (2024). Tackling the outbreak of Nipah virus in Bangladesh amidst COVID-19: a potential threat to public health and actionable measures. Health Sci. Rep..

[61] World Health Organization (WHO). (n.d.). One health. World Health Organization. 2024, from https://www.who.int/health-topics/one-health#tab=tab_1 (accessed 23 October 2024).

[62] Broder C.C., Weir D.L., Reid P.A. (2016). Hendra virus and Nipah virus animal vaccines. Vaccine.

[63] Balarajan Y., Selvaraj S., Subramanian S.V. (2011). Health care and equity in India. Lancet.

[64] Macinko J., Harris M.J. (2011). Brazil's Family Health Strategy: delivering community-based primary care in a universal health system. N. Engl. J. Med..

[65] Joshi R., Cardona M., Iyengar S., Sukumar A., Raju C.R., Raju K. (2006). Chronic diseases now a leading cause of death in rural India: mortality data from the Andhra Pradesh Rural Health Initiative. Int. J. Epidemiol..

[66] Looi L.M., Chua K.B. (2007). Lessons from the Nipah virus outbreak in Malaysia. Malays. J. Pathol..

[67] Olival K.J., Cryan P.M., Amman B.R., Baric R.S., Blehert D.S., Brook C.E. (2017). Possibility for reverse zoonotic transmission of SARS-CoV-2 to free-ranging wildlife: a case study of bats. PLoS Pathog..

[68] Rosales Gerpe M.C. (2023, October 30). Nipah virus outbreak in India: should the US and Canada be concerned?. Clin. Lab.

[69] World Health Organization (2024, February). https://cdn.who.int/media/docs/default-source/searo/whe/wherepib/2024_5_searo_epi_bulletinv3.pdf.

[70] World Health Organization (2024, September 15). https://cdn.who.int/media/docs/default-source/searo/whe/wherepib/2024_19_searo_epi_bulletin.pdf?sfvrsn=f58d473_3.

